# A new species of *Notiobiella* Banks, 1909 from China (Neuroptera, Hemerobiidae), with a key to Chinese species

**DOI:** 10.3897/BDJ.11.e103530

**Published:** 2023-06-02

**Authors:** Yang Zhao, Zhiqi Liu

**Affiliations:** 1 Nanjing Institute of Agricultural Sciences in Jiangsu Hilly Area, Nanjing, China Nanjing Institute of Agricultural Sciences in Jiangsu Hilly Area Nanjing China; 2 China Agricultural University, Beijing, China China Agricultural University Beijing China

**Keywords:** Hemerobiidae, Notiobiellinae, *Notiobiella*, new species, China

## Abstract

**Background:**

*Notiobiella* Banks, 1909 is a green-coloured genus of brown lacewing (Hemerobiidae) that is widely distributed in Central and South America, Africa, southeast Asia, Australia and some south-western Pacific islands. There are approximately 49 species of this genus worldwide, with 10 species recorded from China, including one new species that is described in this paper.

**New information:**

In this paper, we describe a new species, *Notiobiellamaculosa* sp. n. of the genus *Notiobiella* Banks, 1909 from Yunnan Province. The morphological characters of the adults are described in detail and illustrated. A key for identification of adults is also provided. All specimens have been deposited in the Entomological Museum of China Agricultural University (CAU), Beijing.

## Introduction

The genus *Notiobiella* was erected by Banks (1909), based on the type species *Notiobiellaunita* Banks, 1909. *Notiobiella* belongs to the subfamily Notiobiellinae (Nakahara 1960) and is widely distributed in Central and South America, Africa, southeast Asia, Australia and some south-western Pacific islands. *Notiobiella* is recognisable by the presence of a combination of forewing characters, i.e. anterior radial trace bearing two prestigmal "radial sectors", CuP forked proximal to crossvein 2cua-cup and prestigmal subcostal space no wider than adjacent subcostal vein (sometimes slightly wider adjacent to crossvein 1sc-r). Moreover, the male, characterised by the presence of the eversible phallolingua of the gonarcus, is diagnostic (Oswald 1993).

Presently, about 49 species of Notobiella were described (Oswald 1993, Oswald 1999, Monserrat 2000, Garzón-Orduña et al. 2016, Zhao 2016, Oswald 2023), ten of which are recorded from China, including the new species herein.

## Materials and methods

The specimens were examined under a SZ760 stereomicroscope. Images of wings were taken with a Nikon EOS D3200 digital camera attached to the stereomicroscope. The terminalia were observed under a Leica DM2500 compound microscope. Descriptions are based on observations under the stereomicroscope with direct light on specimens preserved in 75% ethyl alcohol. The abdominal apex with the genitalia was cut off, heated in 10% sodium hydroxide for 10-20 minutes and then transferred to an excavated slide containing glycerine. After examination, it was transferred to fresh 75% ethyl alcohol and stored in a microvial.

Wing venation terminology follows Oswald (1993) and Makarkin and Wedmann (2009). Terminology of genitalia follows Oswald (1993).

Abbreviations: 7S, 8S, 9S, sternite; 7T, 8T, 9T, tergite; CuA, anterior cubitus; CuP, posterior cubitus; Ect, ectoproct; egps, extragonopons; egs, extragonarcus; ehgs, extrahemigonarcus; gl, gonapophyses laterales; h, proximal humeral trace; hgs, hemigonarcus; igps, intragonopons; ihgs, intrahemigonarcus; M, Media; m-cu, mediocubital crossvein; med, mediuncus; orb#, oblique radial branch of anterior radial trace; tl, terminal lobe.

## Taxon treatments

### Notiobiella maculosa sp. n.

B4D3A6A1-C040-5930-89B2-8B2BFFEE14C7

1CBAC14C-25A6-497E-A2E3-D2C330AEBB04

*Notiobiella* Banks, 1909; Type species: *Notiobiellaunita* Banks, 1909

#### Materials

- **Type status:** Holotype. **Occurrence:** recordedBy: Yang Zhao; individualID: ZY.N.M1 (CAU); individualCount: 1; sex: male; lifeStage: adult; occurrenceID: 6A424D45-C605-5ABC-BBFB-DA2AA5BDCAE7; **Taxon:** kingdom: Animalia; phylum: Arthropoda; class: Insecta; order: Neuroptera; family: Hemerobiidae; genus: Notiobiella; specificEpithet: *maculosa*; taxonRank: Species; **Location:** continent: Asia; country: China; countryCode: CN; stateProvince: Yunnan; county: Hekou Yao Autonomous County; locality: Binlangzhai Reservoir; verbatimElevation: 315 m; verbatimLatitude: 22°53.484′N; verbatimLongitude: 103°9.60′E; **Identification:** identifiedBy: Yang Zhao; Zhiqi Liu; **Event:** year: 2009; month: 5; day: 21; **Record Level:** language: en; basisOfRecord: PreservedSpecimen
- **Type status:** Paratype. **Occurrence:** recordedBy: Yang Zhao; individualID: ZY.N.M2-3 (CAU); individualCount: 2; sex: 1 male, 1 female; lifeStage: adult; occurrenceID: EACC547A-FD82-51D4-8588-1DF623E35E97; **Taxon:** kingdom: Animalia; phylum: Arthropoda; class: Insecta; order: Neuroptera; family: Hemerobiidae; genus: Notiobiella; specificEpithet: *maculosa*; taxonRank: Species; **Location:** continent: Asia; country: China; countryCode: CN; stateProvince: Yunnan; county: Hekou Yao Autonomous County; locality: Binlangzhai Reservoir; verbatimLocality: 315 m; verbatimLatitude: 22°53.484’N; verbatimLongitude: 103°9.60'E; **Identification:** identifiedBy: Yang Zhao; Zhiqi Liu; **Event:** year: 2009; month: 5; day: 21; **Record Level:** language: en; basisOfRecord: PreservedSpecimen
- **Type status:** Paratype. **Occurrence:** recordedBy: Yang Zhao; individualID: ZY.N.M4 (CAU); individualCount: 1; sex: 1 female; lifeStage: adult; occurrenceID: D8BDB5DA-9453-521E-82F2-3B6F8B55946A; **Taxon:** kingdom: Animalia; phylum: Arthropoda; class: Insect; order: Neuroptera; family: Hemerobiidae; genus: Notiobiella; specificEpithet: *maculosa*; taxonRank: Species; **Location:** continent: Asia; country: China; countryCode: CN; stateProvince: Yunnan; county: Mengla County; locality: Wangtianshu; verbatimElevation: 690 m; verbatimLatitude: 32°6.46'N; verbatimLongitude: 118°8.24'E; **Identification:** identifiedBy: Yang Zhao; Zhiqi Liu; **Event:** year: 2009; month: 5; day: 9; **Record Level:** language: en; basisOfRecord: PreservedSpecimen

#### Description

Body length 3.2-4.2 mm (n = 4). Forewing length 4.8-5.8 mm, width 2.1-2.8 mm. Hind-wing length 3.2-4.2 mm, width 1.2-2.8 mm.

Head yellowish-brown. Semicircular brown patten present near the fore margin of each antennal socket and a triangular brown spot between the hind margin of antennal sockets. Frons and mandible brown, last segment of maxillary and labial palpi brown. Antenna amber, with more than forty flagellomeres. Eye reddish-brown with metallic lustre. Thorax fawn, with brown longitudinal stripes along the sides of pronotum. Lateral margin of mesothorax and metathorax scutum light brown, darker than around. Legs yellowish-brown with no spots.

**Figure 1.**
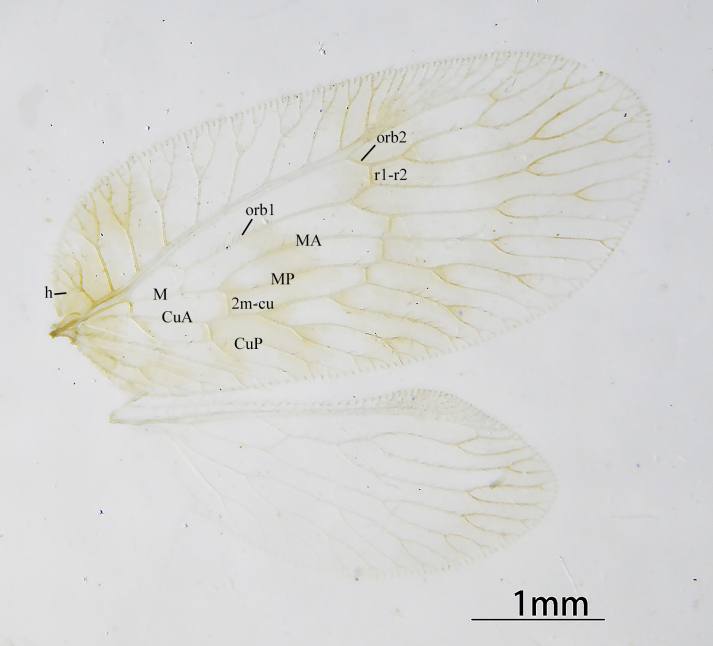
*Notiobiellamaculosa* sp. n., wings.

Forewing (Fig. 1) oval, yellowish-brown. Four brown stripes parallel to gradated series from basal to lateral. Veins yellowish-brown and transparent. Base of costal space wider at the end and proximal humeral trace present. Anterior radial trace bearing two ORBs, with two to four secondary branches respectively; r1-r2 present after ORB1 fork. M with two branches, which have two secondary branches, respectively; 2r-m before M fork. Three m-cu present, with 2m-cu located after M fork and before CuA fork. CuA with four branches and CuP with two; 2cua-cup present after the fork of CuP. One gradated series with four crossveins. Hind-wing oval, pale yellow, hyaline and immaculate; veins pale yellow. Rs with four branches, 2r-rs corssvein present. M forked into two branches, with two secondary branches, respectively. Cu simple. Gradated series with only one crossvein.

**Figure 2.**
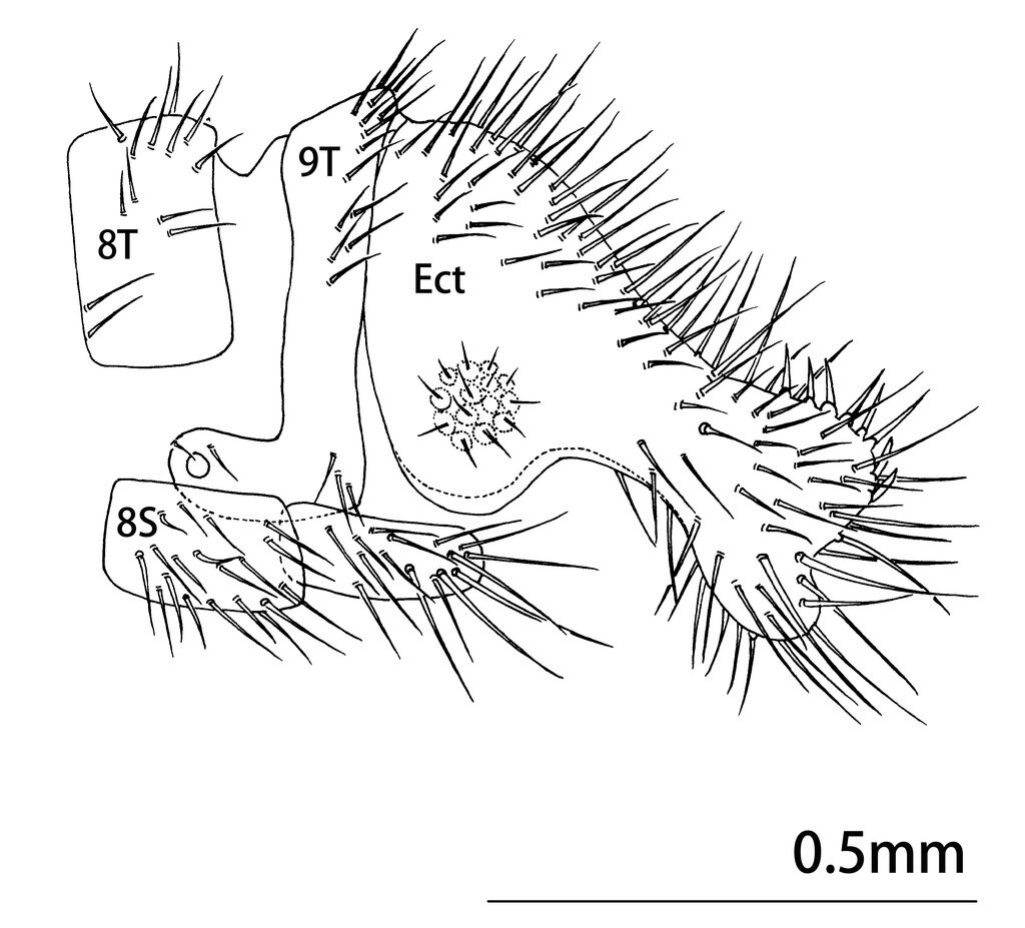
*Notiobiellamaculosa* sp. n., male terminalia, lateral view.

**Figure 3.**
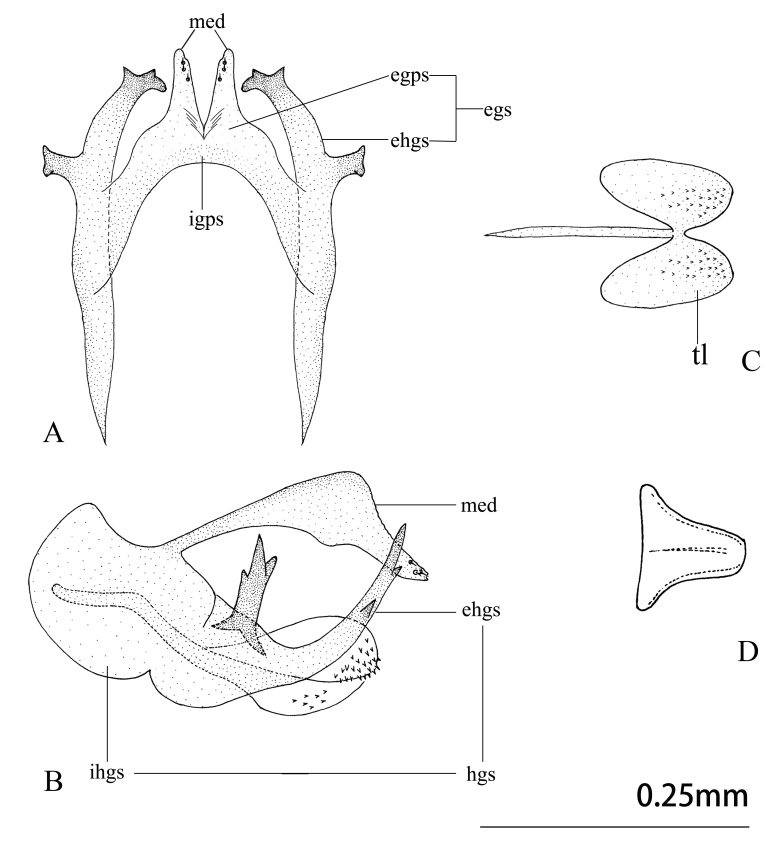
*Notiobiellamaculosa* sp. n. **A** Gonarcus, dorsal view; **B** Ditto, lateral view; **C** Parabaculum, dorsal view; **D** Hypandrium internum, ventral view.

Abdomen yellowish-brown, pilose. Male terminalia (Figs 2, 3). 8^th^ tergite and sternite approximately rectangular in lateral view. Anteroventral edge of 9^th^ tergite bent forwards, including spiracles. 9^th^ stemite small, rectagular in lateral view. Ectoproct developed, broadened basally, narrowing at mid-length and the expanding laterally in lateral view; caudal margin indented with six or seven large spines present along posterodorsal edge. A gap present between the intragonopons and extragonopons; base of mediuncus broad with a median pair of large spines with small spines present on inner surface; extrahemigonarcus bent upwards terminally hook-shaped and bifurcated; extrahemigonarcus with a large thorn on outer surface, ending into three forks; both extrahemigonarcus connected with membrane, with small spines on the surface. Parabaculum simple, with terminal lobe ovoid in dorsal view, slightly prickly. Hypandrium internum in shape of a trapezium in dorsal view.

**Figure 4.**
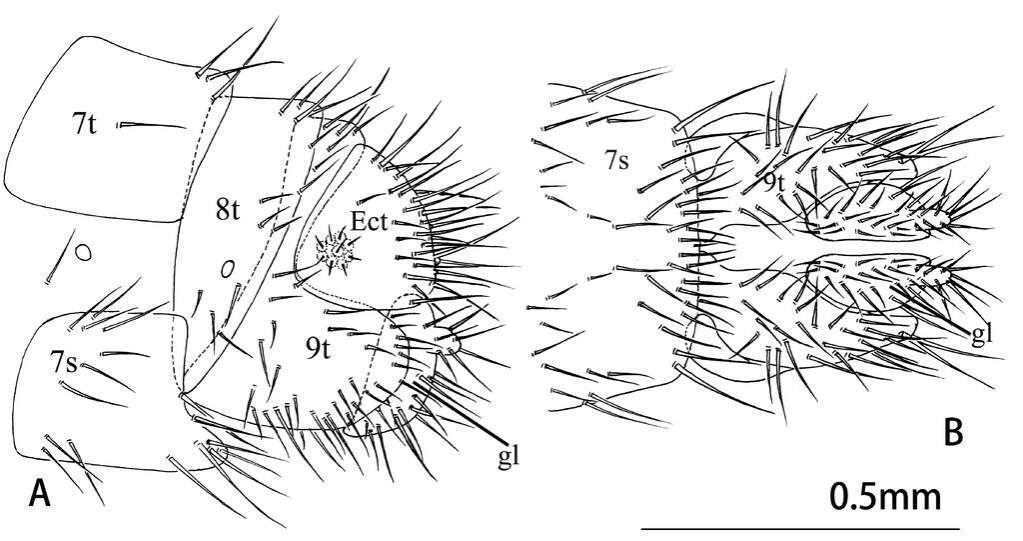
*Notiobiellamaculosa* sp. n. **A** Female terminalia, lateral view; **B** Ditto, ventral view.

Female terminalia (Fig. 4). 8^th^ tergite healed with 8^th^ sternite, approximately triangular from lateral view. 9^th^ tergite vaguely "L" shaped in lateral view, hind margin almost aligned with posterior margin of ectoproct. Lateral gonapophyses approximately semicircular in lateral view, hind margin slightly longer than posterior edge of ectoproct, with stylus. Posterior margin of ectoproct rounded in lateral view. Subgenitale absent.

#### Diagnosis

The species is characterised by the presence of a brown stripe present along the costal veinlet at the base of forewing and four brown stripes parallel to the gradated series running from the base to the lateral margin. Male: ectoproct developed, median section narrowed and posterior margin expanded in lateral view; the centre of the posteral edge depressed and six or seven large spines present along posterodorsal margin. Female: 9^th^ tergite slightly "L" shaped in lateral view; subgenital absent.

#### Etymology

The specific epithet is a Latin adjective, *maculosus*, i.e. spotted, referring to the obvious spots in forewing.

#### Distribution

China (Yunnan Province).

#### Taxon discussion

**Figure 5.**
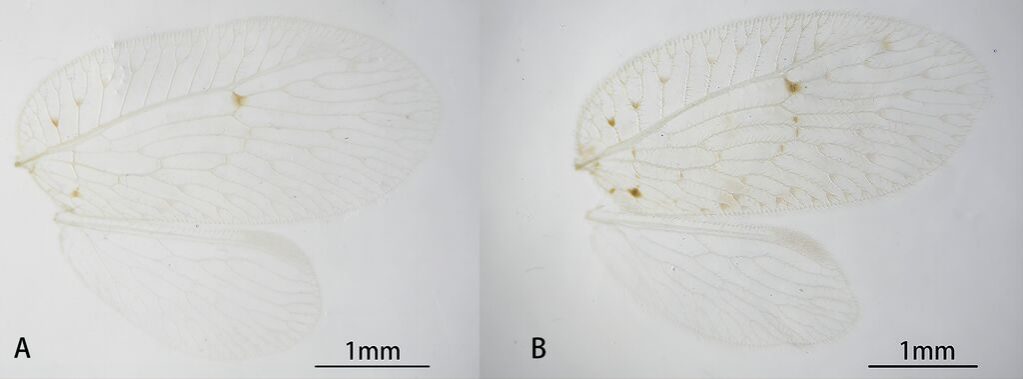
Wings. **A** *Notiobiellasubstellata* Yang, 1999; **B** *Notiobiellastellata* Nakahara, 1966.

**Figure 6.**
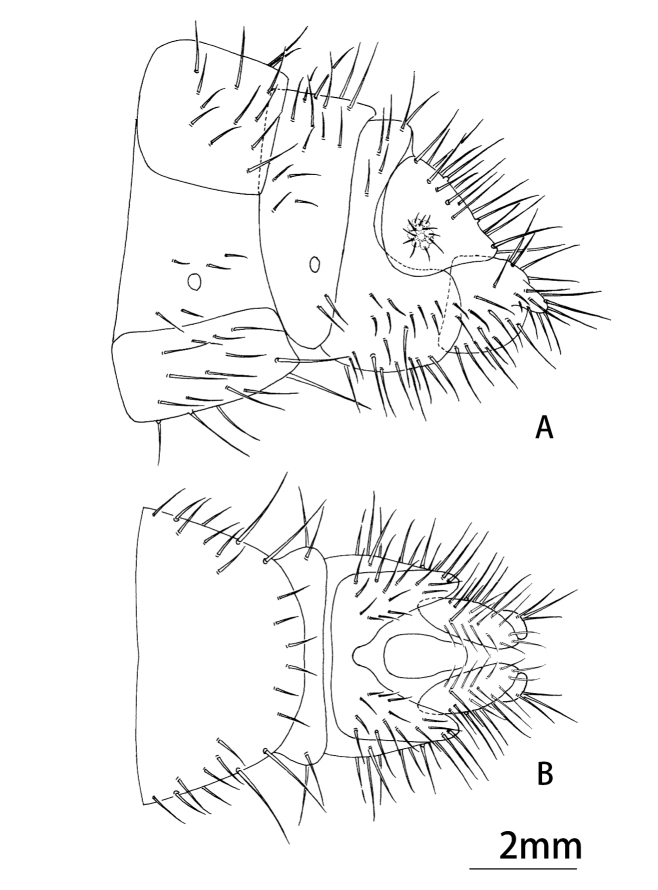
*Notiobiellasubstellata* Yang, 1999. **A** Female terminalia, lateral view; **B** Ditto, ventral view.

**Figure 7.**
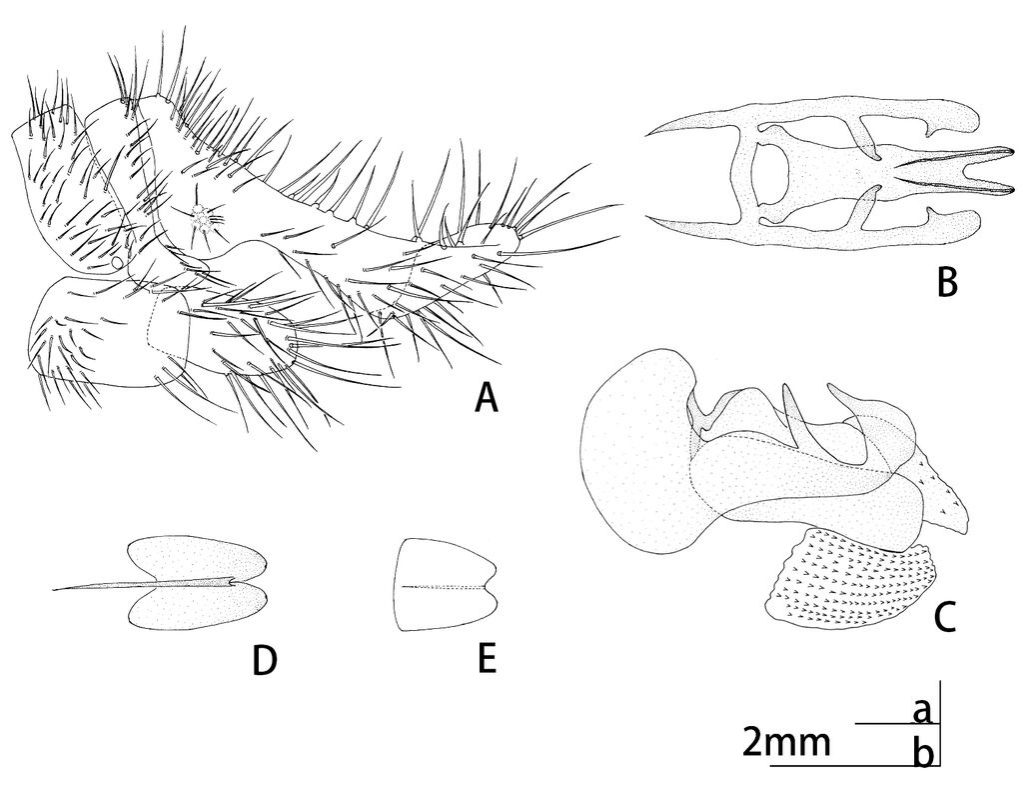
*Notiobiellastellata* Nakahara, 1966. **A** male terminalia, lateral view; **B** Gonarcus, dorsal view; **C** Ditto, lateral view; **D** Parabaculum, dorsal view; **E** Hypandrium internum, ventral view. (Scale bars A:a) .

The new species differs from closely-related species by the presence of spots and stripes in the forewing. *N.maculosa* sp. nov. is similar to *N.substellata* (Fig. 5A) as both species have a spot at crossvein r1-r2 in the forewing, though it can be easily distinguished from the latter by the presence of brown stripes along the costal veinlet and parallel to the gradated series in the forewing. Moreover, the female of *N.maculosa* sp. nov. is devoid of subgenitale, while it is present in *N.substellata* (Fig. 6). The new species is also easily distinguished from *N.stellata* (Fig. 5B) by the 9^th^ tergite bent forwards and the posterodorsal edge of the ectoproct with several large spines. In *N.stellata* (Fig. 7), the 9^th^ tergite is slightly bent backwards and the ectroproct is not provided with large spines.

## Identification Keys

### Key to the species of *Notiobiella* from China

**Table:** 

1	Spot present at crossvein r1-r2 in forewing	2
–	Spot absent at crossvein r1-r2 in forewing	6
2	Spot present at the fork of costal veinlet in basal forewing	3
–	Spot absent at the fork of costal veinlet in basal forewing	5
3	Brown stripe present along the costal veinlet in basal forewing; four brown stripes parallel to the gradated series from basal to lateral; subgenitale absent	*N.maculosa* sp. n.
–	Brown stripe absent along the costal veinlet in basal forewing, only spot present at the fork; no brown stripes parallel to the gradated series; subgenitale present	4
4	Brown spot only present at the fork of basal two costal veinlet; small brown spot only present at crossvein r1-r2 and cua-cup in forewing	*N.substellata* Yang
–	Brown spot present at the fork of every costal veinlet; small brown spot present at every crossvein in forewing	*N.stellata* Nakahara
5	Two brown stripes parallel to the gradated series from basal to lateral in forewing	*N.subolivacea* Nakahara
–	No brown stripes parallel to the gradated series in forewing	*N.sanxiana* Yang
6	Spot present at the fork of costal veinlet in basal forewing	7
–	Spot absent at the fork of costal veinlet in basal forewing	8
7	Three to five taupe round spots present between the basal costal veinlet in forewing	*N.hainana* Yang & Liu
–	No taupe round spots present between the basal costal veinlet in forewing	*N.gloriosa* Navás
8	Pterostigma obviously red in both forewing and hind-wing	*N.ochracea* Nakahara
–	Pterostigma not red in both forewing and hind-wing	9
9	Tumour-like protrusion present at both lateral margin of pronotum, respectively	*N.lichicola* Yang & Liu
–	No tumour-like protrusion present at lateral margin of pronotum	*N.pterostigma* Yang

## Supplementary Material

XML Treatment for Notiobiella maculosa
